# MSC Exosomes Containing Valproic Acid Promote Wound Healing by Modulating Inflammation and Angiogenesis

**DOI:** 10.3390/molecules29174281

**Published:** 2024-09-09

**Authors:** Yujie Mu, Xiaona Zhang, Linfeng Zhang, Ruting Luo, Yin Zhang, Min Wang

**Affiliations:** School of Light Industry Science and Engineering, Beijing Technology and Business University, Beijing 100048, China; muyujie5576@163.com (Y.M.); zhangxn1210@163.com (X.Z.); zhanglf29@163.com (L.Z.); lrt18948322588@163.com (R.L.); zoey2023@163.com (Y.Z.)

**Keywords:** umbilical cord mesenchymal stem cell exosomes, valproic acid, wound healing, angiogenesis, skin regeneration

## Abstract

Purpose: Chronic wounds that are difficult to heal pose a major challenge for clinicians and researchers. Currently, common treatment methods focus on isolating the wound from the outside world, relying on the tissue at the wound site to grow and heal unaided. Umbilical cord mesenchymal stem cell (MSC) exosomes can promote wound healing by enhancing new blood vessel growth at the wound site. Valproic acid (VPA) reduces the inflammatory response and acts on macrophages to accelerate wound closure. In this study, VPA was loaded into umbilical cord MSC exosomes to form a drug carrier exosome (VPA-EXO) with the aim of investigating the effect of VPA-EXO on wound healing. Methods: This study first isolated and obtained umbilical cord MSC exosomes, then added VPA to the exosomes and explored the ability of VPA-EXO to promote the proliferation and migration of human skin fibroblasts (HSFs) and human umbilical vein endothelial cells (HUVECs), as well as the ability to promote the angiogenesis of HUVECs, by using scratch, Transwell, and angiogenesis assays. An in vitro cell model was established and treated with VPA-EXO, and the expression levels of inflammation and pro-angiogenesis-related proteins and genes were examined using Western blot and qRT-PCR. The therapeutic effect of VPA-EXO on promoting wound healing in a whole skin wound model was investigated using image analysis of the wound site, H&E staining, and immunohistochemical staining experiments in a mouse wound model. Results: The in vitro model showed that VPA-EXO effectively promoted the proliferation and migration of human skin fibroblast cells and human umbilical vein endothelial cells; significantly inhibited the expression of MMP-9, IL-1β, IL-8, TNF-α, and PG-E2; and promoted the expression of vascular endothelial growth factors. In the mouse wound model, VPA-EXO reduced inflammation at the wound site, accelerated wound healing, and significantly increased the collagen content of tissue at the wound site. Conclusions: As a complex with dual efficacy in simultaneously promoting tissue regeneration and inhibiting inflammation, VPA-EXO has potential applications in tissue wound healing and vascular regeneration. In future studies, we will further investigate the mechanism of action and application scenarios of drug-loaded exosome complexes in different types of wound healing and vascular regeneration.

## 1. Introduction

Skin wound healing is a complex biological process that involves keratinocytes, fibroblasts, and endothelial cells. Wounds can be caused by pathological conditions, such as surgery, injury, diabetes, or vascular disease [1]. These injuries are categorized as acute or chronic wounds depending on their underlying cause and consequences. Acute wounds, such as abrasions, bites, and lacerations, tend to heal within two weeks. Disturbances during the wound healing phase can cause delays in healing, leading to chronic wounds, which can persist for longer than three months and may never fully heal. Under certain conditions, burns and bites may develop into chronic infections that last a lifetime, which not only seriously affects the quality of life and physical and mental health of patients, but also increases the treatment cost and burdens the economic situation of patients [2,3]. Patients with diabetes mellitus, vascular disease, aging, and hemoglobinopathies are especially prone to abnormal wound healing, which, if not properly addressed, can lead to wound recurrence and even amputation or death in severe cases [4,5].

Wounds can be divided into hemostatic, inflammatory, proliferative, and maturation stages from wound formation to tissue healing [6,7,8]. The body’s first response to a wound is to constrict injured blood vessels and activate platelets to form a fibrin clot, which blocks blood flow and serves as a scaffold for incoming inflammatory cells [9]. The immune system and immune cells are then activated to combat self and foreign antigens. The onset of angiogenesis marks the end of the inflammatory phase, as endothelial cells proliferate, migrate, and branch into new blood vessels. Following the emergence of the new blood vessels, the existing fibroblasts proliferate and differentiate into myofibroblasts, which pull the edges of the wound together. This causes the microenvironment of the wound to shift from an inflammatory state to a growth state, with re-epithelialization occurring simultaneously [10,11,12,13].

Mesenchymal stem cells (MSCs) are pluripotent mesenchymal cells, and the paracrine effects of MSCs play a role in promoting angiogenesis, proliferation, and the migration of epithelial cells and fibroblasts during the wound healing response [14,15,16,17,18]. Stem cell exosomes can be used as nanoscale membrane-bound vesicular particles with sizes ranging from 30–150 nm [19]. They have the advantages of high stability, low immunogenicity, biocompatibility, and a long circulation time, making them natural drug carriers [20,21]. Exosomes can also remodel the extracellular matrix to deliver signals and molecules to other cells, and their use avoids risks such as the tumorigenicity of stem cells and host rejection. Exosomes can migrate to the site of skin injury to perform their functions, improving skin regeneration and reducing skin scarring through inhibiting inflammation and increasing the growth and differentiation capacity of fibroblasts, epidermal cells, and endothelial cells. Exosomes repair wounds by controlling several steps in the wound healing and regeneration process [14,22]. MSC exosomes contain extracellular matrix, nucleic acids, and multiple growth factors, which can play a therapeutic role [23]. In addition, MSC exosomes can also wrap small-molecule drugs with special functions to become drug-loaded exosomes, which have the dual advantages of both exosomes and drugs and play a synergistic effect.

Valproic acid (VPA) is a histone deacetylase (HDAC) inhibitor with potential protective and reparative effects against acute central nervous system (CNS) injury. Because of its good tolerability and safety, VPA has become the drug of choice for the prevention and treatment of epilepsy [24]. VPA not only mediates nuclear factor kappa-B (NF-κB) to attenuate inflammatory responses, but also down-regulates the production of reactive oxygen species (ROS) [25,26]. VPA can attenuate the expression and activation of matrix metalloprotein-9 (MMP-9) through inhibiting histone deacetylases, hinder the production of inflammatory mediators such as TNF-α and IL-6, and influence the cell cycle, cell differentiation, and apoptosis. VPA can promote skin wound healing through enhancing the activity of keratinocytes in mouse models. Cell activity promotes skin wound healing, and VPA also inhibits the inflammatory activation of macrophages and promotes the macrophage phagocytosis of apoptotic cells, suggesting that VPA can accelerate wound closure by acting on macrophages [27]. Therefore, VPA is considered a safe small-molecule drug with the potential to promote wound repair. In this study, human umbilical cord MSC exosomes loaded with VPA were prepared to explore the therapeutic effect of drug-loaded exosomes in wound healing and their potential mechanism of action, providing new ideas for therapeutic approaches to wound repair.

## 2. Results

### 2.1. Effective Development and Characterization of Exosome-Loaded VPA

**Figure 1:**
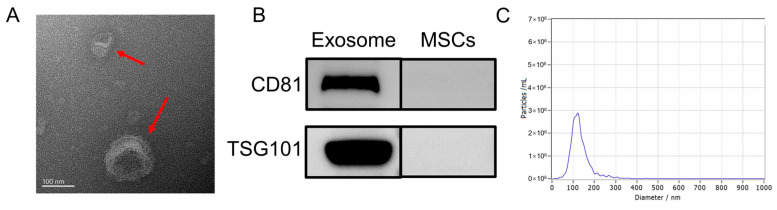
Characterization of UC-MSC-EXO (**A**) TEM morphology of exosomes (Red arrows indicate exosomes) (**B**) Analysis of exosome characteristic markers (**C**) Exosome nanoparticle diameter distribution.

Following previously established protocols, this study isolated the exosomes from HUC-MSCs. Various techniques and analyses were employed to characterize the exosomes. First, TEM was utilized to examine the morphology of UC-MSC-EXO. TEM revealed that the samples exhibited the characteristic cup-shaped structure commonly observed in MSC-derived exosomes (Figure 1A). Next, Western blotting was performed to assess the expression of exosome marker proteins. The analysis revealed the enrichment of tetraspanin proteins (TSG101 and CD81), which are known to participate in exosome transport. Notably, the exosome markers TSG101 and CD81 were detected in the exosome samples through Western blotting, whereas they were not detected in the MSC extracts (Figure 1B). Furthermore, NTA was conducted to determine the size distribution of the extracted particulate matter. Most of the particles fell within the expected size range of 30–150 nm, which was consistent with the typical size range of exosomes (Figure 1C). Collectively, these findings demonstrate the successful isolation of exosomes from HUC-MSCs, characterized by their cup-shaped morphology, appropriate particle size range, and the presence of specific exosome marker proteins. These results demonstrate the isolation of exosomes with high quality and purity from HUC-MSCs.

**Figure 2:**
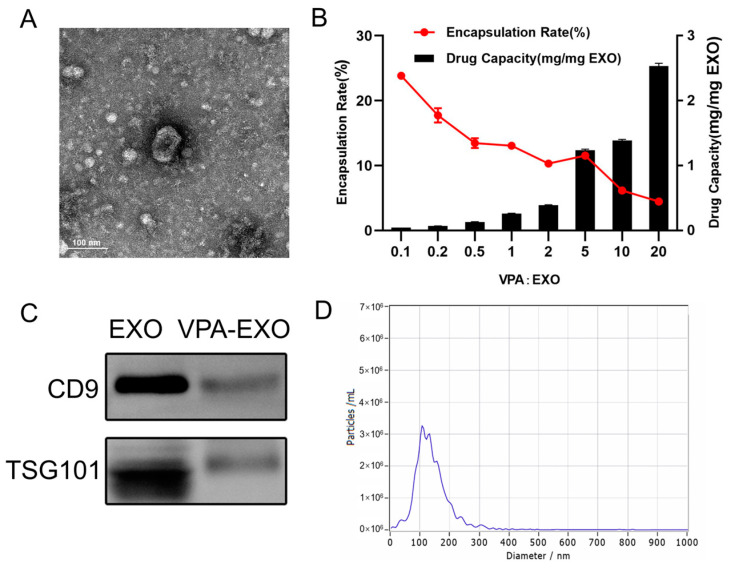
Exosome drug-loading efficiency and characterization of VPA-EXO (**A**) TEM morphology of VPA-EXO (**B**) Exosome drug-loading efficiency at different VPA concentrations (**C**) Characteristic marker analysis of VPA-EXO (**D**) Diameter distribution of VPA-EXO nanoparticles.

In the process of loading VPA into exosomes, this study aimed to determine the appropriate mass ratio of VPA to exosomes for optimal encapsulation. Because no previous reports provided guidance on this matter, a series of experiments were conducted in this study to explore different mass ratios. The initial VPA: exosome mass ratio of 1:10 was gradually increased to 20:1. Interestingly, as the mass ratio increased, the overall trend reflected a decrease in the encapsulation rate but an increase in the amount of drug loading. Specifically, as the mass ratio increased from 1:10 to 2:1, the encapsulation rate gradually decreased, while the amount of drug loading slightly increased. Moreover, when the mass ratio was further increased from 2:1 to 5:1, the amount of drug loading significantly increased, and the encapsulation rate increased to around 11.58%. As the investigation continued, the mass ratio was further increased from 5:1 to 20:1. The drug loading continued to rise, albeit at a slower rate, while the encapsulation rate gradually decreased. Notably, when the mass ratio was 1:10, the highest encapsulation rate was approximately 24.26%. However, the drug loading at this ratio was the lowest. Ultimately, the mass ratio of 5:1 exhibited the most favorable drug loading effect, with an encapsulation rate reaching 11.54% (Figure 2A,B). Consequently, this mass ratio was selected for subsequent experiments. By examining various mass ratios of VPA to exosomes, this work identified the optimal ratio that provided the highest drug loading efficiency while maintaining a satisfactory encapsulation rate. This finding served as a foundation for subsequent experiments.

A similar characterization of VPA-EXO was also performed, as depicted in Figure 2. The TEM analysis demonstrated that VPA-EXO exhibited the characteristic cup-shaped morphology commonly observed in exosomes (Figure 2A). Western blot experiments were conducted to explore the presence of exosome markers in the VPA-EXO samples. The analysis confirmed the presence of the exosome markers CD9 and TSG101 (Figure 2C), which further validated the exosome nature of the VPA-EXO. Similarly, the results of NTA revealed that most of the particle sizes in the extracted particulate matter fell within the expected range of 30–150 nm, aligning with the typical characteristics of exosomes (Figure 2D).

### 2.2. Optimizing the VPA-EXO Concentration for HSF and HUVEC Proliferation and the Wound Healing Assay

**Figure 3:**
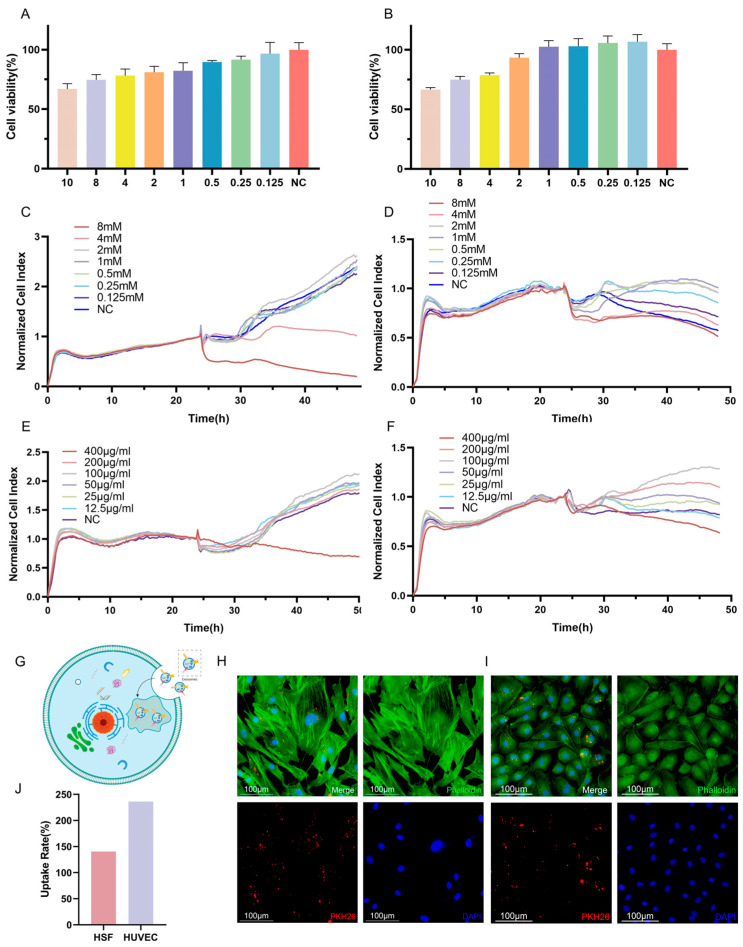
Different concentrations of VPA and VPA-EXO acted on HSF and HUVEC as well as target cell uptake of drug-loaded exosomes (**A**) Cell viability of HSF cells at 24 h (**B**) Cell viability of HUVEC cells at 24 h (**C**) 48 h real-time growth curves of HSF cells under VPA action (**D**) 48 h real-time growth curves of HUVEC cells under VPA action (**E**) VPA EXO effect on HSF cells (**F**) 48 h real-time growth curve of HUVEC cells under the effect of VPA-EXO (**G**) Schematic diagram of cellular uptake of VPA-EXO (**H**) HSF uptake of PKH26-labeled VPA-EXO (**I**) HUVEC uptake of PKH26-labeled VPA-EXO (**J**) HSFs’ and HUVECs’ uptake rate.

To account for the potential adverse effects of VPA on cellular function, experiments were conducted to determine the optimal concentration of VPA for loading into exosomes. Cell viability was assessed as a measure of the impact of VPA on cells. The results obtained from the CCK-8 assay demonstrated a dose-dependent relationship between the VPA concentration and the cell viability, indicating a gradual decrease in cell survival with increasing VPA concentrations. When considering HSF cells, concentrations of VPA lower than 2 mM resulted in a cell survival rate of over 80%, which can be considered a safe concentration (Figure 3A,C,E). Similarly, for HUVECs, concentrations of VPA lower than 2 mM led to a cell survival rate exceeding 90%, indicating a safe concentration. Furthermore, the results obtained from the RTCA revealed that HSF cells were negatively affected when the VPA concentration exceeded 4 mM, resulting in lower cell activity compared to the negative control. Conversely, concentrations of VPA lower than 2 mM resulted in a growth curve for HSF cells that was similar to that of the negative control, indicating a safe concentration. For HUVECs, significant decreases in growth curves were observed when VPA concentrations of 8 mM and 4 mM were applied, with no evident trend of increased cell growth over time. Conversely, concentrations of VPA lower than 2 mM resulted in higher growth curves for HUVECs compared to the negative control, indicating a safe concentration. Considering the comprehensive analysis of the above results, a dosing concentration range of 0.125–2 mM was selected for subsequent experiments as it fell within the safe concentration range based on the observed cell viability and growth curves (Figure 3B,D,F).

To investigate the cellular uptake of VPA-EXO, PKH26 labeling was utilized to track the movement pathway of VPA-EXO. The PKH26-labeled VPA-EXO was co-cultured with HSFs and HUVECs separately. Notably, the PKH26-labeled drug-loaded exosomes were observed within the HSF cells (Figure 3G,H) and HUVECs (Figure 3I), indicating the successful internalization of the drug-loaded exosomes by the cells. To explore the role and underlying mechanism of VPA-EXO in promoting wound healing, HSFs and HUVECs were selected for in vitro cellular experiments. Determining a safe concentration was crucial for subsequent experiments, which was achieved using the RTCA method to establish cell growth curves.

### 2.3. Effect of VPA-EXO on Wound Healing in an In Vitro Model

**Figure 4:**
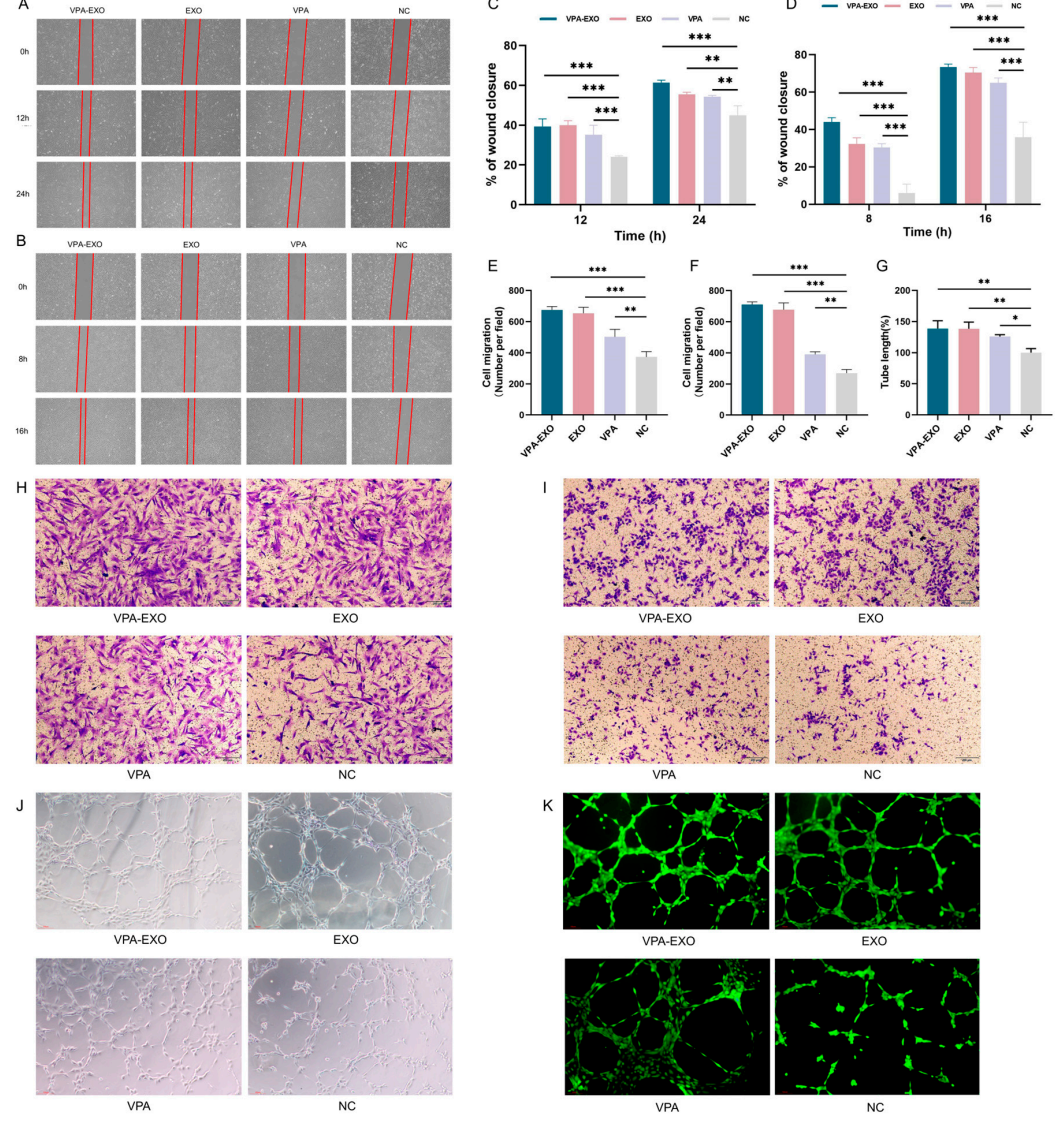
VPA-EXO promotes cell proliferation and migration in vitro (**A**) Optical images of HSF cell scratch assay (**B**) Optical images of HUVEC cell scratch assay (**C**) Quantitative results of HSF cell scratch assay (**D**) Quantitative results of HUVEC cell scratch assay (**E**) Quantitative results of HSF cell Transwell assay (**F**) Quantitative results of HUVEC cell Transwell assay Results (**G**) Quantitative results of HUVEC cell tube-forming assay (**H**) Optical image of HSF cell Transwell assay (**I**) Optical image of HUVEC cell Transwell assay (**J**) Optical image of HUVEC cell tube-forming assay (**K**) Optical image of HUVEC cell fluorescence staining tube-forming assay. (* indicating *p* < 0.05, ** indicating *p* < 0.01, and *** indicating *p* < 0.001).

To investigate the potential effects of VPA-EXO on wound healing using an in vitro model, experiments were conducted to assess the ability of VPA-EXO to regulate cell proliferation and migration. Angiogenesis is an important component of the wound-healing process and the basis of tissue repair. HUVECs are the main cells that make up blood vessels and are essential for the formation of new blood vessels [28,29]. HSF cells have special contractile properties, which play an important role in the generation and remodeling of connective tissues during the skin wound healing period [30]. In this study, a scratch assay and a Transwell assay were conducted to test the ability of VPA-EXO to regulate cell proliferation and migration. A cellular wounding model was constructed in HSFs and HUVECs, and HSFs/HUVECs were randomly divided into experimental and control groups. The experimental groups were treated with VPA-EXO, VPA, and EXO at a concentration of 100 μg/mL and the control group was treated with an equal amount of PBS to observe the effects of VPA-EXO on the proliferation and migration of HSFs and HUVECs and to assess the ability of VPA-EXO to promote wound healing (Figure 4A,B). As confirmed by light microscopy, the migration rate of cells treated with VPA-EXO was significantly higher than that of the control group, and VPA-EXO promoted the proliferation of HSFs and HUVECs at 12 and 24 h, in addition to enhancing the migration ability of cells. (Figure 4H,I).

To further assess their tubule-forming ability, HUVECs were co-cultured with each group of different drugs and observations were conducted using an inverted microscope to examine tubule formation (Figure 4J,K). The results revealed a greater number of tubular structures in HUVECs treated with VPA-EXO compared to the EXO and VPA groups, with the negative control (NC) group exhibiting the lowest formation of vascular network-like structures. Quantitative analysis of the tubule length in each group using Image J software (1.54c) confirmed that the tubule length in the VPA-EXO group was significantly higher than that in the NC group (*p* < 0.01). These findings suggest that VPA-EXO treatment enhances the tubule-forming ability of HUVECs. In summary, the results of these experiments demonstrated that VPA-EXO positively influenced cell proliferation and migration in the context of wound healing using an in vitro model. The scratch assay, Transwell assay, and tubule formation analysis collectively support the possibility that VPA-EXO exhibits wound healing-promoting properties.

### 2.4. Impact of VPA-EXO on the Expression of Key Genes Involved in Wound Healing

To gain insights into the mechanistic modulation of the wound healing process by VPA-EXO, the expression levels of key factors associated with wound healing were further investigated. IL-1β and TNF-α are crucial proinflammatory factors that induce the secretion of various proinflammatory mediators. IL-1β, a significant member of the IL-1 family, exhibits strong proinflammatory activity and stimulates the production of cytokines and chemokines [28]. Similarly, TNF-α is a multifunctional proinflammatory cytokine belonging to the TNF ligand superfamily, and its involvement has been implicated in several diseases [29,30]. TNF-α can bind to specific receptors and activate downstream signaling pathways to modulate the inflammatory response, including chemokines, adhesion molecules, and secondary cytokines. IL-8 and related cytokines are highly effective in initiating the inflammatory response following exposure to inflammatory stimuli, such as IL-8 or TNF-α. These cytokines are produced in cells and tissues in response to infection, inflammation, ischemia, and trauma, and are considered the main contributors to local neutrophil accumulation [31].

**Figure 5:**
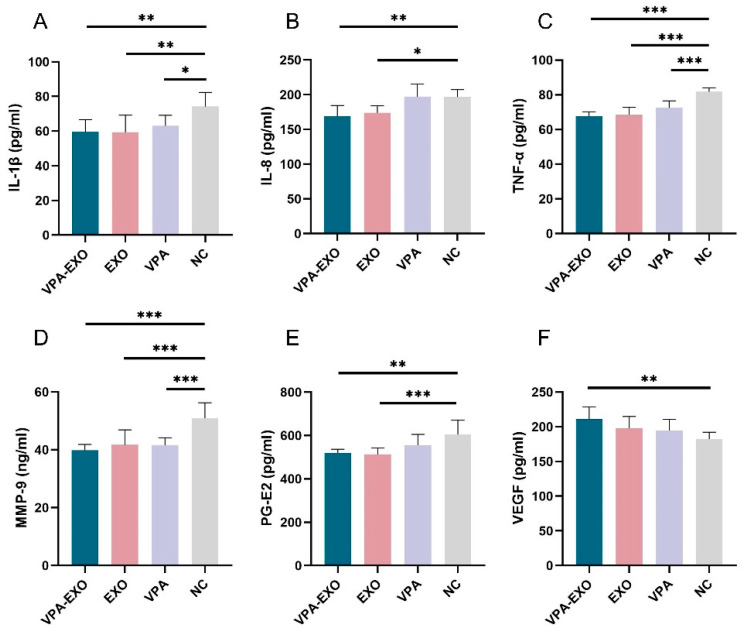
Expression of cytokines by ELISA (**A**) IL-1β (**B**) IL-8 (**C**) TNF-α (**D**) MMP-9 (**E**) PG-E2 (**F**) VEGF. (* indicating *p* < 0.05, ** indicating *p* < 0.01, and *** indicating *p* < 0.001).

To further validate the role of VPA-EXO in the wound healing process, ELISA was employed to measure the secretion of related factors in vascular endothelial cells and fibroblasts. As shown in Figure 5, the ELISA results indicated that compared to the NC group, VPA-EXO, EXO, and VPA all reduced the expression of IL-1β, IL-8, TNF-α, MMP-9, and PG-E2, while promoting the expression of VEGF. The expression levels of IL-1β, IL-8, TNF-α, MMP-9, and PG-E2 in the VPA-EXO group were significantly lower than those in the NC group, indicating that the strongest inhibitory effect was achieved. Additionally, vascular endothelial growth factors (VEGFs) belong to the platelet-derived growth factor supergene family and play a central role in regulating angiogenesis [32]. VEGF-A, a major factor in angiogenesis, binds to two tyrosine kinase (TK) receptors, VEGFR-1 (Flt-1) and VEGFR-2 (KDR/Flk-1), and regulates endothelial cell functions including proliferation, migration, and vascular permeability. Angiopoietin (Ang), as a pro-angiogenic factor, participates in the growth, development, and remodeling of blood vessels. Ang binds to Tie2, its corresponding receptor, and the Ang/Tie2 signaling pathway is a recently reported angiogenic pathway that participates in the regulation of angiogenesis and development. The expression of VEGFs was significantly elevated. These findings indicate that VPA-EXO can inhibit proinflammatory factors and promote angiogenic factors, thus exerting a positive influence on wound healing.

**Figure 6:**
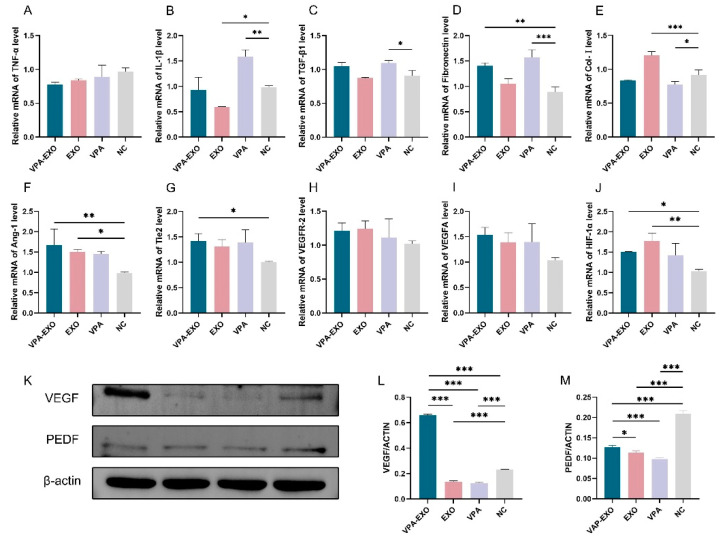
Wound healing-related factors mRNA expression levels and protein immunization results. (**A**) TNF-α (**B**) IL-1β (**C**) TGF-β1 (**D**) Fibronectin (**E**) Col-І (**F**) Ang-1 (**G**) Tie2 (**H**) VEGFR-2 (**I**) VEGFA (**J**) HIF-1α (**K**) Relative protein expression of VEGF and PEDF (**L**) Quantitative analysis of relative protein expression of VEGF (**M**) Quantitative analysis of relative protein expression of PEDF. (* indicating *p* < 0.05, ** indicating *p* < 0.01, and *** indicating *p* < 0.001).

qRT-PCR was employed to examine the expression levels of several angiogenesis-related genes at the mRNA level to elucidate the effect of VPA-EXO on the expression of wound-healing-related genes. The results of qRT-PCR showed that the expression of TNF-α and IL-1β mRNA in VPA-EXO-treated HSF cells was lower than that in the NC group, and the expression of TGF-β1 and fibronectin was significantly elevated (Figure 6). The Ang-1, Tie2, and HIF-1α angiogenesis-related factors were significantly elevated in HUVECs, while the expression of VEGFR-2 and VEGFA was not significantly elevated (Figure 6). The results indicated that VPA-EXO could inhibit inflammation and promote angiogenesis after acting on vascular endothelial cells and fibroblasts.

VEGF, as one of the most potent angiogenesis-stimulating factors and vascular permeability factors, is essential in the process of vascular neogenesis. Pigment epithelium-derived factor (PEDF) is an endogenous inhibitor of angiogenesis that exhibits differentiation and neuroprotective activities as well as antiangiogenic activity and can inhibit endothelial cell migration in a dose-dependent manner in vitro [33,34]. To further investigate the effect of VPA-EXO on angiogenesis during wound healing, the expression levels of proteins involved in the regulation of angiogenesis (e.g., VEGF and PEDF) were determined using Western blot. The results revealed that the expression of VEGF significantly increased (*p* < 0.01) and the expression of PEDF significantly decreased (*p* < 0.01) following VPA-EXO treatment.

Collectively, these findings support the role of VPA-EXO in promoting and regulating angiogenesis and reducing inflammation, highlighting its potential therapeutic effects in the wound healing process.

### 2.5. VPA-EXO Improves Skin Wound Healing in Mice

**Figure 7:**
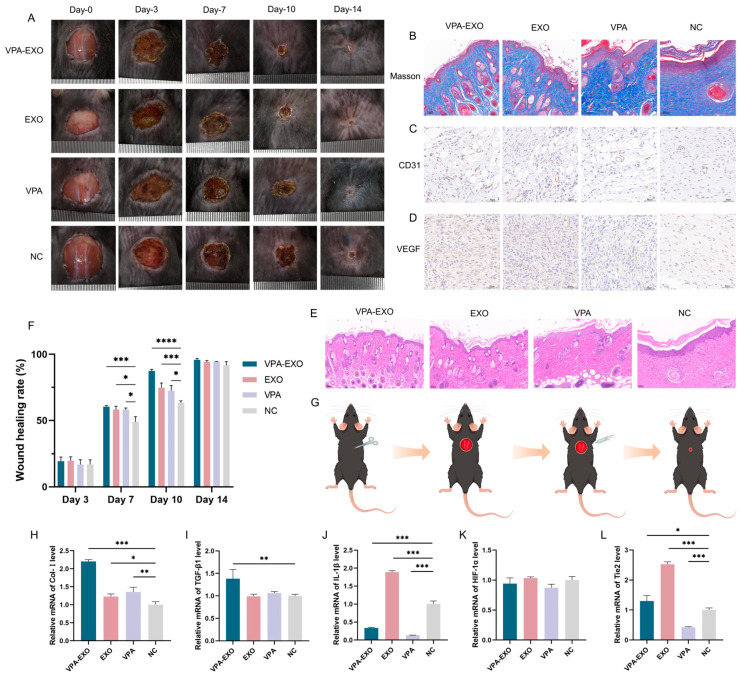
Effects of VPA-EXO on skin wound healing and expression of skin tissue-associated factors at the wound site in mice (**A**) Photographs of skin wound healing in mice (**C**) Immunohistochemical analysis of CD31 (**D**) Immunohistochemical analysis of VEGF (**E**) Optical image of H&E staining of the wound on day 14 after treatment (**B**) Optical image of Masson staining of the wound on day 14 after treatment (**F**) Mice skin wound healing rate (**G**) Mice treatment (**H**) Col-І (**I**) TGFβ-1 (**J**) IL-1β (**K**) HIF-1α (**L**) Tie2. (* indicating *p* < 0.05, ** indicating *p* < 0.01, *** indicating *p* < 0.001 and **** indicating *p* < 0.0001).

To further substantiate the beneficial effects of VPA-EXO on the wound healing process, experiments were conducted using a mouse wound healing model to evaluate the impact of VPA-EXOs. Specifically, full-thickness skin wounds were created on the backs of mice, and these wounds were treated with VPA-EXO, EXO, VPA, or PBS. The wound healing process was categorized into four stages: hemostasis, inflammatory response, proliferation, and remodeling. Representative images of the wound area for each group were captured on days 0, 3, 7, 10, and 14 after treatment (Figure 7A).

According to the results of the in vivo experiments, some inflammatory reactions were observed in all groups on day 3, with no significant difference in the rate of wound healing among the groups. As depicted in Figure 7A, by day 7 after treatment the inflammatory phase had subsided and the wound surface had begun to shrink, resulting in the formation of thin, dry, dark brown scabs across all groups. On the 10th day of treatment, the VPA-EXO group exhibited the most pronounced wound healing, displaying the smallest wound area among the groups. By the 14th day, the skin wounds in all groups had healed, but the VPA-EXO treatment group showed significantly faster wound closure compared to the control group (Figure 7A).

To assess the efficacy of wound repair, H&E and Masson staining were performed to evaluate tissue re-epithelialization and collagen formation. On the 14th day of the experiment, the mice in the VPA-EXO group exhibited the most favorable results in terms of tissue re-epithelialization and the formation of granulation tissue (Figure 7E). The VPA-EXO-treated wounds had a thin layer of new epidermis and had transformed into normal tissue, indicating the effective promotion of epithelial tissue formation. Collagen staining on day 14 revealed abundant, compact, and well-aligned collagen fibers in the VPA-EXO-treated wounds (Figure 7B). Histological analysis of the wound site revealed that the VPA-EXO, EXO, and VPA treatments all exhibited therapeutic effects compared to the control group. The wound repair time was shortened and the recovery of the skin structure was accelerated after VPA-EXO treatment (Figure 7A,B,E), which was consistent with the in vitro experimental results. These findings suggest that VPA-EXO treatment accelerates the healing of skin wounds in mice.

Angiogenesis, a crucial physiological process in wound repair, was evaluated using immunohistochemical staining for VEGF and CD31 in wound tissues on the 14th day of treatment. The results demonstrated that all three experimental groups promoted the expression of CD31 and VEGF. The VPA-EXO group exhibited a higher positive expression of CD31 and an increased expression of VEGF compared to the control group, implying the effective promotion of angiogenesis during the wound-healing process (Figure 7C,D).

qRT-PCR analysis was further conducted to investigate the expression levels of key genes involved in wound healing, including Col-І, TGFβ-1, IL-1β, HIF-1α, and Tie2. In the VPA-EXO group, the skin tissues exhibited significantly elevated expression levels of Col-І, TGFβ-1, and Tie2 (Figure 7H,I,L). Conversely, the expression level of IL-1β was significantly decreased in the VPA-EXO group compared to the control group (Figure 7J). Although the expression of Ang-1 increased in the VPA-EXO group, the difference was not statistically significant compared to the control. Additionally, there was no significant difference in the expression of HIF-1α between the treatment groups and the control group (Figure 7K).

In conclusion, these comprehensive experiments provide evidence that VPA-EXO plays a significant role in promoting physiological wound healing in a mouse model. This effect is achieved through the modulation of the expression of key genes, tissue re-epithelialization, collagen formation, and angiogenesis.

## 3. Discussion

The treatment of wound healing has long been a key research area in medicine. Cell therapy through the introduction of biologically active stem cells is not only expected to promote wound healing but also may improve therapeutic efficacy, especially for difficult-to-heal or chronic wounds. The use of stem-cell-derived exosomes in wound healing therapy has received increasing attention and has become a research hotspot in recent years [35]. In the inflammatory stage of the wound, exosomes can promote macrophage activation and tissue repair. Exosomes have high pro-angiogenic and cell growth factor secretion activity, which can promote cell proliferation and migration, inhibit inflammation, and enhance vascular neogenesis in the process of wound healing. Exosomes have achieved remarkable efficacy in the treatment of tumors and immune diseases and have shown great potential for application in clinical trials, representing an ideal solution for cell-free therapy in the field of regenerative medicine [36,37]. In addition, some therapeutic drugs still have limitations in their application. For example, large-molecule drugs are not easily absorbed in the human body, are prone to cytotoxicity, and cannot promote the healing of trauma sites, limiting their clinical application. Exosomes derived from MSCs have certain advantages, such as natural targeting, low immunogenicity, and their ability to be easily frozen and transported. As a result, MSC-derived exosomes are considered to exhibit potential as drug delivery carriers. Exosome-based drug delivery systems can overcome biological barriers and thus provide excellent therapeutic effects. Exosomes can be used as carriers for many types of drugs, including nucleic acids, proteins, and even small-molecule drugs. Using exosomes as drug carriers can increase the solubility, stability, and bioavailability of drugs and improve drug delivery efficiency. This means that MSC exosomes can not only be used in treatment but can also act as drug carriers, thereby improving the drug treatment effect. Drug-loaded exosomes have great therapeutic potential in tissue repair and regeneration. VPA, as a histone deacetylase inhibitor, exhibits anti-inflammatory, antioxidant, and anti-tumor activities, especially for the nerves, with potential protective and restorative effects. VPA can up-regulate the expression of neurotrophic factors, which means that VPA can promote not only angiogenesis but also nerve regeneration, thus further accelerating wound closure [38]. In the near future, VPA can be utilized in combination with other pro-nerve repair drugs, enabling VPA to play a greater role.

Although the experimental results demonstrate that VPA-EXO has good prospects in wound repair, more clinical practice is needed to verify its effect and safety in actual treatment, which will be favorable for future clinical applications. In addition, in terms of the drug-loading method, indirect drug-loading is commonly used but it also suffers from insufficient characterization and low loading efficiency, while the direct drug-loading method is relatively simple and requires further development. In terms of isolation and purification, the classical ultracentrifugation method requires costly equipment, and the other methods need to be improved in terms of isolation effectiveness and integrity. In the future, it is necessary to further investigate the molecular mechanism of drug-loaded exosomes, as well as factors including the source, isolation methods, culture conditions, drug-loaded methods, and drug delivery schemes. In addition, research in this field should move towards the realization of standardized large-scale production as soon as possible. We believe that drug-loaded exosomes will not only be effective in the field of wound healing and dermatological diseases, but will also enable further breakthroughs in other systemic diseases in the future.

## 4. Materials and Methods

### 4.1. MSC-EXO Preparation and Extraction

MSC cells with a fusion rate of 80–90% were washed with phosphate-buffered saline (PBS) two to three times, added to a serum-free medium for stem cells, and cultured for another 24 h. Twenty-four hours later, the cell supernatant was collected and centrifuged at 3000× *g* for 10 min, after which the supernatant was taken and exosome concentration solution (ECS reagent) was added according to the selected ratio, mixed well, and then allowed to stand for 14 h at 4 °C. After another round of centrifugation (10,000× *g* for 1 h), the supernatant was discarded and PBS was added for resuspension, followed by another round of centrifugation (12,000× *g* for 2 min). The supernatant was then transferred to the upper chamber of the exosome purification filter (EPF column). Centrifugation (3000× *g* for 10 min) was performed a final time and the liquid at the bottom of the EPF column was collected and stored at −80 °C for subsequent use.

### 4.2. MSC-EXO Identification

#### 4.2.1. Transmission Electron Microscopy (TEM)

Each exosome sample (10 μL) was dropped onto a copper grid so that the hydrophilic side was in contact with the liquid, and the excess liquid was absorbed with filter paper. The sample was washed with ultrapure water and then stained with drops of uranyl acetate (UA) staining solution for 30 s. The sample was allowed to dry naturally. The samples were placed under a transmission electron microscope and the morphology and size of exosomes were observed.

#### 4.2.2. Nanoparticle Tracking Analysis (NTA)

The particle size and concentration of exosomes were detected using a nanoparticle tracking analyzer (NanoSight NS500, Zetaview, Dusseldorf, Germany). Calibration was performed before use and the samples were washed with PBS three times. PBS-diluted samples were added to the nanopore to measure and analyze the particle size and concentration of exosomes.

#### 4.2.3. Western Blot Assay

The lysis solution was added to the sample proportionally and left on ice for 3–5 min. The sample was centrifuged at 12,000× *g* for 5 min, the supernatant was collected, the sample buffer was added according to the specified proportion, and the sample was heated for 10 min. The gelator was placed into the electrophoresis tank, the electrophoresis solution was added, the sample was dotted in, and electrophoresis was initiated. When the bromophenol blue was about 1 cm from the bottom, electrophoresis was terminated and the membrane was transferred. During the membrane transfer process, the membrane transfer equipment was cooled down. Tris buffered saline with tween (TBST) was added to wash the membrane, skimmed milk was added, and the membrane was closed for 30 min at 25 °C. After washing, the prepared primary antibodies TSG101 (1:1000) and CD81 (1:1000) were added and incubation proceeded at 4 °C overnight. After washing the membrane with TBST, the secondary antibody was added, incubation was performed at room temperature for 2 h, and the membrane was washed again. Well-mixed ECL solution (1:1) was added and allowed to react fully. After 1–2 min, strip images were obtained using a chemiluminescence image analysis system.

### 4.3. Preparation and Characterization of Drug-Loaded Exosomes

The exosomes and VPA mixture were loaded with the drug using an ultrasonic cell crusher. After sonication, the mixture was incubated at 37 °C for 60 min, and the free drug was isolated via centrifugation at 5000× *g* for 10 min using an ultrafiltration tube. The drug-loading efficiency of exosomes was determined using liquid chromatography with mobile phase A as acetonitrile: 0.1 mol/L sodium acetate (4.2 g of anhydrous sodium acetate was taken and 30 mL of acetonitrile was dissolved in 970 mL of water) solution (3:97), with the pH adjusted to 6.5 using acetic acid. Mobile phase B consisted of acetonitrile: water (4:1). The flow rate was 1.0 mL/min and the detection wavelength was 210 nm. TEM observations, the NTA assay, and the Western blot assay were also performed on the drug-loaded exosomes as described above.

### 4.4. Cell Culture for In Vitro Models

Human umbilical cord mesenchymal stem cells (HUC-MSCs) were purchased from SAIYE (Guangzhou, China) Biotechnology Co., Ltd. and cultured to the third generation using serum-free medium for stem cells. Human skin fibroblasts (HSFs) were purchased from Peking Union Medical College Hospital (Beijing, China) and cultured to the ninth generation using Dulbecco’s modified eagle medium (DMEM) high-sugar medium supplemented with fetal bovine serum and penicillin–streptomycin. Human umbilical vein endothelial cells (HUVECs) were purchased from Henan Provincial Industrial Microbial Strain Engineering and Technology Research Center (Nanyang, China) and cultured to the third generation using endothelial cell culture medium for experiments. The cell lines were cultured in a cell culture incubator (37 °C, 5% CO_2_).

### 4.5. Cytotoxicity Assay

HSF cells and HUVECs in the logarithmic growth phase were collected and the cell density was adjusted to 8 × 10^4^ cells/mL. The cells were inoculated into 16-well and 96-well plates and incubated at 37 °C and 5% CO_2_ for 24 h. The 16-well plates were placed into the real-time cell analysis (RTCA, Agilent, Santa Clara, CA, USA) system for real-time monitoring, and the 96-well plates were subjected to the Cell Counting Kit-8 (CCK-8) assay. Intervention treatments using different concentrations of VPA were conducted for 24 h, while control wells were set up. After replacing the original culture medium with culture medium containing 10 μL of CCK-8 solution, the cells were incubated for 2 h and the optical density value at 450 nm was determined using an enzyme-labeling instrument.

### 4.6. VPA-EXO to Promote Skin Wound Healing In Vitro

#### 4.6.1. Uptake of Drug-Loaded Exosomes

The PKH26-labeled VPA-EXO was utilized in co-culture with HSFs or HUVECs for 24 h. The cells were washed with pre-cooled PBS, fixed with 4% paraformaldehyde for 10 min, washed three times with PBS, blocked using an anti-fluorescent bursting agent, and placed under a fluorescence microscope for observations.

#### 4.6.2. Migration Ability of HSFs and HUVECs

HSF cells and HUVECs in the logarithmic growth phase were collected, the cell density was adjusted to 5 × 10^5^ cells/mL, the two-well inserts from the scratch assay were placed into six-well plates, and 70 μL of cell suspension was added to the inserts. The cells were cultured in an incubator at 37 °C and 5% CO_2_ for 24 h. One milliliter each of different drugs was added for treatment. Culture was performed in an incubator and pictures were collected at the same position at 0, 4, 8, 12, and 24 h of culture. The width and area of the scratched area were measured using Image J software, and the cell migration rate at each period was calculated as (scratched area at 0 h − scratched area at x h)/scratched area at 0 h.

#### 4.6.3. Transwell Assay of the Invasive Ability of HSFs and HUVECs

A cell invasion assay was performed in 24-well plates in a Transwell filtration chamber. First, 600 μL of serum-free medium containing VPA-EXO, EXO, and VPA was added to the lower chamber and an equal amount of serum-free medium containing PBS was added to the control group. Finally, 200 μL of cell suspension was added to the upper chamber, which was incubated for 20–24 h at 37 °C with 5% CO_2_, fixed in 4% tissue cell fixative for 20 min, and stained using 0.1% crystal violet for 5 min.

#### 4.6.4. Tube-Forming Ability of HUVECs

The Ibidi angiogenesis slides were removed and 10 μL Matrigel was added to each well. After standing in the incubator for 30 min and waiting for the matrix gel to solidify, 50 μL of cell suspension with a concentration of 2 × 10^5^ cells/mL was added to the upper well of the angiogenesis slides and images were collected at regular intervals in accordance with the growth rate of the cells. Angiogenesis was observed using a microscope. Then, 50 μL of Calcein AM solution at a concentration of 1 μM was added and the cells were incubated at 37 °C for 30 min away from light, washed with PBS, and observed using a fluorescence microscope for immunofluorescence imaging. The tubule lengths of each experimental group were statistically analyzed using Image J software to examine the effects of different drugs on the experimental results.

#### 4.6.5. Examination of Cytokines in the Supernatants of HSFs and HUVECs

The levels of interleukin 1β (IL-1β), interleukin 8 (IL-8), tumor necrosis factor (TNF-α), matrix metalloproteinase 9 (MMP-9), and prostaglandin E2 (PG-E2) in the supernatant of HSF cells and vascular endothelial growth factor (VEGF) in the supernatant of HUVECs were detected using enzyme-linked immunosorbent assay (ELISA). The expression of relevant cytokine mRNA in HUVECs was detected using quantitative reverse transcription polymerase chain reaction (qRT-PCR). RNA was extracted from HUVECs and 15 μL of nuclease-free water was added to solubilize the RNA. Incubation was performed at 55 °C for 5 min and each sample was diluted to a concentration of 100–500 ng/μL. The total samples collected from the experimental group and the blank group were quantified using a microspectrophotometer. cDNA was quantified using a microspectrophotometer, the reaction system was configured, and the corresponding primers were designed according to the target genes, after which the corresponding PCR program was initiated. The expression of VEGF and PEDF in HUVECs was detected via Western blot, and the experimental procedure was the same as that described above.

#### 4.6.6. In Vivo Study of VPA-EXO to Promote Skin Wound Healing

Forty-four male C57BL/6 mice aged 6–8 weeks were used to construct a whole-layer skin injury model and randomly divided into VPA-EXO, EXO, VPA, and PBS groups. The mice were acclimatized for 7 days. After fasting for 12 h before the experiment and anesthesia, the backs of the mice were shaved. The depilated area was about 2.5 cm × 2.5 cm, and one circular wound measuring 1 cm in diameter was constructed on the backs of all the mice to ensure adequate exfoliation from the skin, dermis, and muscularis propria. The date of model establishment was recorded as day 0. Each mouse was injected subcutaneously with 50 μL of VPA-EXO (EXO/VPA/PBS) into the four midpoints of the wound margin. Wounds were observed on postoperative days 0, 3, 7, 10, and 14 to assess wound healing. On the 14th postoperative day, the trauma tissues were sampled, and the skin tissues at the wound were excised, flash-frozen in liquid nitrogen, and stored at −80 °C. The frozen skin tissue was rapidly ground until no visible particles were present. The total RNA was extracted from the tissues and the expression levels of the related factors Col-І, IL-1β, TGF-β1, HIF-1α, Tie2, and Ang-1 in the skin tissues were detected using qRT-PCR following the method described above. The skin tissues of the wounds of mice on days 7 and 14 were taken and embedded, and then they were subjected to Hematoxylin and Eosin(H&E) and Masson staining. Wound skin tissues collected on day 14 were subjected to immunohistochemistry to detect the expression levels of CD31 and VEGF. The histologic changes of the wounds were observed under the microscope.

### 4.7. Statistical Analysis

One-way analysis of variance (ANOVA) was applied to the results obtained using GraphPad Prism 9 to analyze the differences between the groups, with *p* < 0.05 considered statistically significant, * indicating *p* < 0.05, ** indicating *p* < 0.01, and *** indicating *p* < 0.001.

## 5. Conclusions

Umbilical cord MSC exosomes can promote skin wound healing through inhibiting inflammation and promoting collagen production and angiogenesis. To study the therapeutic effect and mechanism of action of exosomes combined with VPA in wound healing, this study successfully loaded VPA into exosomes derived from HUC-MSCs. The VPA-EXO prepared using ultrasound technology exhibited the morphological characteristics of exosomes, with particle size mainly distributed from 30–150 nm and a membrane structure. In addition, the relative expression levels of CD9 and TSG101 proteins, which are characteristic surface markers of exosomes, were positive. When the mass ratio of VPA to exosome was 5:1, the drug-loading effect was the best and the encapsulation rate could reach 11.54%.

In vitro investigations showed that fibroblasts and vascular endothelial cells were able to uptake PKH26-labeled VPA-EXO, and in vitro cellular experiments (including cell proliferation and migration assays and tubule formation) demonstrated that VPA-EXO effectively promoted the proliferation, migration, and angiogenic effects of HSFs and HUVECs. ELISA and qRT-PCR were performed on a number of cytokines, the expression of inflammatory regulatory factors, and angiogenesis-related factors, demonstrating that VPA-EXO could significantly inhibit the expression of MMP-9, IL-1β, IL-8, TNF-α, and PG-E2 and promote the expression of VEGFs. In addition, VPA-EXO significantly inhibited the expression of inflammation-related cytokines IL-1β and TNF-α mRNA and induced the up-regulated expression of Ang-1, Tie2, VEGFR-2, VEGFA, and HIF-1α mRNA in HUVECs. VPA-EXO promoted the expression of VEGF protein and inhibited the expression of PEDF protein. These findings suggest that VPA-EXO regulates wound healing through inhibiting inflammation and enhancing angiogenesis, thus promoting wound healing.

In a mouse skin trauma model, VPA-EXO significantly reduced the inflammatory manifestations of skin wounds, increased the wound healing rate, reduced the inflammatory factors in skin tissues, significantly increased the collagen content, accelerated angiogenesis and the re-epithelialization of wounds, thereby shortening the time of wound repair, and accelerated the recovery of the skin structure. These results were consistent with the in vitro experiments and demonstrated that VPA-EXO treatment accelerated skin healing in mice. VPA-EXO shows potential for utilization in wound healing and vascular regeneration, and the mechanism of action of drug-loaded exosomes in promoting wound healing and vascular regeneration can be further investigated to expand their therapeutic role in wound healing.

## Data Availability

The original contributions presented in the study are included in the article; further inquires can be directed to the corresponding authors.
